# Assessment of Oral Health-Related Quality of Life and Its Associated Factors among the Young Adults of Saudi Arabia: A Multicenter Study

**DOI:** 10.1155/2022/5945518

**Published:** 2022-01-13

**Authors:** Ashokkumar Thirunavukkarasu, Abdulaziz M. Alotaibi, Ahmed H. Al-Hazmi, Bashayer F. ALruwaili, Mohammad A. Alomair, Waleed H. Alshaman, Amjed M. Alkhamis

**Affiliations:** ^1^Department Community and Family Medicine, Division of Community Medicine, College of Medicine, Jouf University, Sakaka 72388, Saudi Arabia; ^2^Department of Public Health, Ministry of Health, Riyadh 11176, Saudi Arabia; ^3^Department Community and Family Medicine, Division of Family Medicine, College of Medicine, Jouf University, Sakaka 72388, Saudi Arabia; ^4^Department of Academic Affairs and Training, Ministry of Health, Tabuk Region 47312, Saudi Arabia; ^5^Department of Public Health, Ministry of Health, Tabuk Region 47312, Saudi Arabia

## Abstract

Oral health-related quality of life (OHRQoL) is an essential indicator of people's overall health and health-related quality of life. Poor oral health and OHRQoL among young adults lead to numerous negative consequences and an increased burden on the healthcare system. The present study is aimed at assessing the OHRQoL among the young adults of Saudi Arabia, identifying self-rated oral health, and determining the relationship between sociodemographic and lifestyle factors with the OHRQoL. The present analytical cross-sectional survey was conducted among 1152 health and non-health-related college university students from three randomly selected universities. The OHRQoL was evaluated using the validated Arabic version of the oral health impact profile-14 questionnaire (OHIP-14). Of the population studied, one-fourth of the participants (24.9%) reported poor or fair oral health, and the highest OHIP-14 score was found in the domains of physical pain (4.14), followed by psychological discomfort (4.07). Logistic regression analysis revealed that the poor oral health category was significantly associated with male gender (ref: female: adjusted OR (AOR) = 1.89, 95%CI = 1.23–2.94, *p* = 0.004), daily smokers (ref: nonsmokers: AOR = 3.47, 95%CI = 1.97–4.82, *p* < 0.001), chocolate and candies intake more than once a day (ref: never; AOR = 1.54, 95%CI = 1.10–2.19, *p* = 0.034), and did not seek periodical dental care (ref: periodic dental care received: AOR = 2.23, 95%CI = 1.53‐2.86, *p* = 0.002). The present study revealed the factors associated with poor OHRQoL. The concerned authorities should consider the implementation of periodic dental checkups for university students, especially for the high-risk group. Furthermore, it is recommended to have regular health education programs that will help to change the student's lifestyle and poor oral health behaviors.

## 1. Introduction

Health is “a state of complete physical, mental, and social well-being, not merely the absence of disease and infirmity” as defined by the World Health Organization (WHO) [1]. Oral health (OH) is an essential indicator of people's general health and is closely associated with overall health and health-related quality of life (HRQoL) [2, 3]. HRQoL is an appropriate index for assessing people's overall health and the effect of health conditions on the quality of life [4]. Understanding the health and quality of life leads us to understand the concept of oral health-related quality of life (OHRQoL) [5]. OHRQoL represents the subjective experience of symptoms related to oral conditions that impact the well-being of an individual. The OHRQoL uses patient-centered outcome measures to identify the impact of OH on aspects of everyday life regarding social, psychological, and functional well-being [5, 6]. Poor OH and OHRQoL among people lead to numerous negative consequences, including low self-esteem, depression, decreased performance in daily activities, lack of social interaction, and an increased burden on the healthcare system [3, 7].

Over the past decades, a set of psychometric instruments have been developed to assess OHRQoL [8, 9]. The OH impact profile (OHIP) questionnaire is commonly used to measure OHRQoL in children, adults, and dentate elderly people [10, 11]. The short version of the OHIP includes 14 items (OHIP-14), which are based on Locker's conceptual model to measure OH [10]. These elements represent the consequences of oral diseases and the negative impact they have on OHRQoL. The validity and reliability of OHIP-14 have been shown in many studies, and the instrument has been translated and validated into several languages including Arabic language [9, 12–15]. The factors that affect self-reported OH are not clear, but it has been suggested that oral diseases have a detrimental effect on subjective OH, and that effect is likely to be greater at younger ages. Studies reported that young and middle-aged adults report worse OH than older adults, although oral problems tend to increase with age [16, 17].

In a study conducted by Wei et al. among Japanese students, they reported that young university students are in a dynamic transition period of growth and development that bridges adolescence to adulthood (people in the community) [18]. Many of them start to live away from their homes for the first time in their lives, which can adversely affect their health, lifestyle, and behavior. As a result of these physiological and social changes, their oral health behaviors and their clinical status can quickly deteriorate [18, 19]. Furthermore, poor oral health behaviors, such as high sugar consumption and inadequate brushing habits, may lead to adverse effects on OHRQoL [19, 20].

In the Kingdom of Saudi Arabia (KSA), some authors have attempted to identify the OHRQoL in different settings. Most of the studies that assessed OHRQoL were among patients with a dental problem or elderly participants [14, 17]. Despite the evidence on the importance of OHRQoL assessment among apparently healthy young adults and university students, there is limited data available in the KSA. Considering the necessity of having regional data in this category population, this study was planned to assess the OHRQoL among the young adults of the KSA by using the OHIP-14 questionnaire. The present study is also aimed at determining the relationship between OHRQoL and self-rated oral health among them and to find the association between sociodemographic and lifestyle factors with OHRQoL.

## 2. Participants and Methods

### 2.1. Study Description

This multicenter cross-sectional study was carried out among students (aged 18-25) from different colleges (health- and non-health-related colleges) from three universities of the KSA, namely, Jouf University, Northern Border University, and King Saud University.

### 2.2. Sample Size Estimation

The minimum required participants for this study was estimated based on Cochran's formula for sample size estimation, *n* = *z*^2^*pq*/*e*^2^. In this formula, *z* = 1.96 in the 95% confidence interval, *e* is in the 5% error margin, *p* is the expected proportion, and *q* is 1*p*. Since studies that assessed OHRQoL among the KSA population had depicted huge variations in prevalence in this subject, we have taken a 50% population proportion (*p*) to obtain a maximum sample size. After applying all the values in Cochran's formula, the estimated minimum required sample for the current survey was 384. The present study included 384 students from each university, and the total estimated sample size was 1152 (3 × 384).

### 2.3. Sampling Method

The required number of sample participants was selected by a multistage probability proportional to size (PPS) sampling technique. In this technique, first, two health science colleges and two other colleges were randomly selected using the lot method from each of the three universities. Only a college with both sections (boys and girls) is included. In the following stages, the required number of participants from each college was selected according to gender and year of the students. Finally, the systematic random sampling method was used based on students' university identification numbers to select participants from each year of education.

### 2.4. Data Collection Method

We have begun the data collection process after ethical approvals obtained from concerned authorities and other necessary administrative permissions. The data collector contacted the selected student during the self-directed learning period with the help of their class leader. After briefly explaining about the survey and getting informed consent, the students filled the data collection form (Google form) in the data collector's electronic device (mobile, tab, or laptop). Only the principal investigator was authorized to access the Google form of the current survey. Students who were not willing to participate or were unavailable during data collection were considered nonrespondents and were replaced by the next student according to the university identification number.

### 2.5. Data Collection Tool

This research data were collected by using an open-source, structured, and validated self-administered Arabic questionnaire [15] consisting of three parts. The first part inquired about sociodemographic and oral health-related behaviors such as intake of candies, sugary drinks, and brushing teeth. The second part inquired about the participants' perceptions of their oral health by asking through a question “What is your perception of your oral health?,” and the responses were recorded from poor to excellent. Finally, we assessed OHRQoL through OHIP-14. The OHIP-14 has 14 questions related to the evaluation of OHRQoL. The responses were recorded in a 5-point Likert scale ranging from never to very often (score is 0 to 4). Then, we calculated the total scores of all domains, and a higher score indicated poor OHRQoL. The highest possible total score of all the OHIP-14 domains is 56 (14 × 4). Furthermore, OHRQoL was further categorized into good, for whom score was less than 60% of the total possible score (<35), and poor for whom score was more than or equal to 60% of the total possible score (≥35). The OHIP-14 assesses seven domains in a broad area ranging from functional and social to psychological discomfort caused by their oral health conditions [9, 11]. The present study used an open-source Arabic version of the OHIP-14 tool, validated by Habashneh et al. among Jordanian adults (satisfactory Cronbach's alpha value > 70) [15]. A pilot study was carried out among 30 health and non-health science college students in our region with the adapted Arabic questionnaire. The Cronbach alpha value of the pilot study was 0.89, which exhibited good internal consistency. Hence, the research team proceeded to collect the main study data using this standard and validated Arabic questionnaire.

### 2.6. Data Analysis

The Microsoft Excel sheet downloaded from Google form was exported to Statistical Package for the Social Sciences (SPSS) version 21. Then, we recoded all the variables in SPSS as per the predefined data coding sheet for further analysis. Descriptive data from this study were presented as frequency and proportion (*n*; %) for qualitative variables, while quantitative variables were shown as the mean ± SD for age in sociodemographic characteristics. Initially, the research team performed the Shapiro-Wilk test for normality assumption. The Kruskal-Wallis test was applied to find the association between perceived oral health status and OHIP-14 scores. The binomial logistic regression (enter method) analysis was executed to determine the relationship between oral health category status and sociodemographic factors, lifestyle factors, and oral health behaviors. In this enter method, the adjusted covariables were age in years, gender, college type, year of education, smoking status, carbonated drink intake, chocolates, candies consumption, brushing count, and periodic dentist care. This study's statistical tests were two-tailed, and a *p* value less than 0.05 was set as statistically significant value.

## 3. Results

Of the 1152 studied participants, the mean (SD) age was 20.98 (1.9), 51.6% were females, the majority (57.5%) were from non-health-related colleges, and more than three-fourth (80.6%) were nonsmokers. Almost a third of the participants were taking carbonated drinks daily (34.8%) and consuming chocolates and candies (34.4%) daily. Regarding oral health-related behaviors, 59.4% of the participants were brushing teeth once a day and a majority (60.1%) of them never visited dental healthcare providers periodically (Table 1).

The participants' responses in each item of the OHIP-14 questionnaire are presented in Table 2. The majority (76.4%) of the study participants never had trouble pronouncing words, two-third (67.1) of the participants never had difficulty doing their usual jobs, and 6.5% of students were self-conscious due to their oral health.

**Figure 1:**
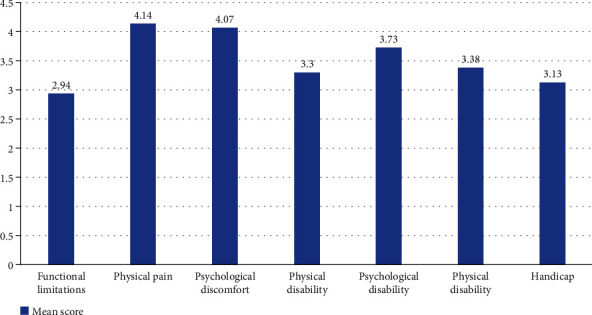
Mean score distribution in different OHIP-14 domains.

Figure 1 shows the distribution of mean scores in seven domains of the OHIP-14. Of the participants studied, the highest score was found for physical pain (4.14), followed by psychological discomfort (4.07) and psychological disability (3.73). The mean total score of all seven domains was 24.69 ± 5.2.

Table 3 shows that almost one-third (34.4%) of the participants reported good oral health. Furthermore, the combined OHIP-14 scores the self-rated oral health were assessed by the Kruskal-Wallis (nonparametric) test. Of the sample studied, there was a significant association between self-rated oral health (*p* < 0.001) and pain or discomfort in teeth or gum or mouth (*p* = 0.012) with the OHIP-14 scores.

**Figure 2:**
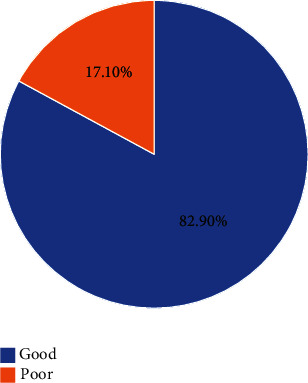
Overall OHRQoL category (*n* = 1152).

The participants were further classified into good (<35 of total score) and poor (≥35 of total score) OHRQoL categories. Of the 1152 university students who participated, 197 (17.1%) were in the poor oral health category and 955 (82.9%) were in the good OHRQoL categories (Figure 2). These categories were used for logistic regression analysis.

The results of binomial logistic regression analysis that was done to find the relationship between oral health category status with the sociodemographic characters, lifestyle factors, and oral health behaviors are presented in Table 4. The poor oral health category was significantly associated with male gender (ref: female: AOR = 1.89, 95%CI = 1.23–2.94, *p* = 0.004), daily smokers (ref: nonsmokers: AOR = 3.47, 95%CI = 1.97–4.82, *p* < 0.001), chocolate and candies intake more than once a day (ref: never; AOR = 1.54, 95% CI = 1.10–2.19, *p* = 0.034), and not seeing the dental care provider periodically (ref: periodic dental care: AOR = 2.23, 95%CI = 1.53-2.86, *p* = 0.002).

## 4. Discussion

“World oral health day” is observed annually on the 20th of March by the World Dental Federation to reduce the burden of oral diseases. Their campaign theme 2021-2023 focused on inspiring the people on the importance of oral health for its positive effect on general health, well-being, and overall healthy life [21]. This reiterates the importance of assessing OHRQoL among young adults for preventive measures to improve their overall health, as they will be the future of a country.

Previous researchers worldwide stated that self-rated OH is one of the critical links and predictors of the general health status of the public [22, 23]. The present study findings revealed that one-fourth (24.9%) of the participants perceived their OH status as either poor or fair. A survey conducted by Drachev et al. among Russian university students also revealed similar findings [24]. In contrast, a study done by Moreas et al. has shown a higher proportion of poor self-rated health. This difference in results could be described due to the study settings, inclusion criteria, and methods used. The present study included young adults of both sexes studying at the university, while the latter included only women from a Brazilian community [25]. The present study revealed a positive association between the OHIP-14 scores and self-oral rated health (*p* < 0.001). This study finding is supported by researches of Verhulst et al. and Drachev et al. [23, 24]. Those studies also reported a positive association between perceived poor oral health status and the OHIP-14 scores. These findings again confirm that self-rated oral health is one of the strongest predictors of the OHRQoL and general health.

The results of the current study indicated that the highest OHIP score was found in physical pain, followed by the domains of psychological discomfort and psychological disability domains. Similarly, a study conducted in Jazan city of Saudi Arabia also found that physical pain and psychological discomfort domains had higher OHIP scores than the rest of the domains [14]. Interestingly, a study done by Papaioannou et al. among the Greek population revealed that high scores were determined in functional limitation, physical pain, and handicap domains [26]. This dissimilarity could be justified by the variations in the incorporation of the survey participants. The current study included university students from health and other colleges, while Papaioannou et al. surveyed the adults from rural and urban communities.

On assessing the association between sociodemographic characteristics with the poor OHRQoL, the present study found that male participants had a significantly higher rate of poor OHRQoL (AOR = 1.89, 95%CI = 1.23–2.94, *p* = 0.004) than females. However, previously published studies around the world reported different findings. For example, a study done in China by Lu et al. did not find any differences between genders and OHRQoL [27], and a study by Drachev et al. in Russia reported a higher rate of low OHRQoL [24].

This study revealed that poor OHRQoL was significantly higher among the daily smokers (AOR = 3.47, 95%CI = 1.97–4.82, *p* < 0.001). Similar to the current study findings, most previous studies also reported a significant association between smokers and poor OHRQoL [28, 29]. Numerous mechanisms explain this striking association between smoking and oral health, including decreased blood flow, increased local edema, and inflammation [30, 31].

Although there are many debates about the periodic screening interval for preventive dental care, the American Dental Association suggested dental screening and evaluation every six months. The current study reported that 60.1% of the participants did not seek dental care providers periodically. The poor OHRQoL was significantly higher among the students who did not visit dental healthcare providers regularly (AOR = 2.23, 95%CI = 1.53 − 2.86, *p* = 0.002). Some researchers have previously evaluated the effectiveness of periodic dental screening [32, 33]. In the KSA, the Ministry of Health has established a dental screening program among schoolchildren. However, no structured dental screening program is implemented in university facilities for students.

Despite the best efforts made by the present survey team on this study with a standard methodology, certain constraints are to be noted while reading the findings of the current survey. Firstly, this survey is a questionnaire-based and self-reported. Hence, recall bias, exaggerated responses, and selection bias are to be considered while interpreting the findings of this survey. Secondly, this cross-sectional study attempted to find the association, and not the causation, between the variables.

## 5. Conclusions

The study findings suggest that self-rated poor oral health is significantly associated with OHIP-14 scores. The present study revealed the factors associated with poor OHRQoL. Physical pain and psychological discomfort were the most common domain with high OHIP scores. The concerned authorities should consider the implementation of periodic dental checkups for university students, especially for the high-risk group. Furthermore, regular health education programs that help change the student's lifestyle and oral health behaviors must be arranged. Finally, an exploratory multicenter study is warranted that compares OHRQoL with the actual oral status of the students.

## Figures and Tables

**Table 1:** Distribution of study participants according to sociodemographic characteristics, lifestyle factors, and oral health behaviors.

Characteristics	Number	%
Age (mean ± SD)	20.98 ± 1.9	
Gender Male Female	594 558	51.6 48.4
College type Healthcare-related college Other colleges	490 662	42.5 57.5
Year of education First Second Third Fourth Fifth	303 240 233 154 222	26.3 20.8 20.2 13.4 19.3
Smoking/shisha habits No Yes: daily Yes: rarely	928 156 68	80.6 13.5 5.9
Carbonated drink consumption Never Once a day Seldom/rarely	152 401 599	13.2 34.8 52.0
Consumption of chocolates and candies Never Seldom/rarely Once daily More than once a day	45 525 397 185	3.9 45.6 34.4 16.1
Brushing teeth per day Once Twice or more	684 468	59.4 40.6
Visit to the dental provider periodically (every 6 months) Yes No	460 692	39.9 60.1

**Table 2:** Frequency of responses in each domain of OHIP-14. The data shown below are frequency and proportion; *n* (%).

Domains	Items	Never	Hardly ever	Occasionally	Fairly often	Very often
Functional limitations	Trouble pronouncing words	880 (76.4)	106 (9.2)	108 (9.4)	39 (3.4)	19 (1.6)
	Taste worsened	808 (70.1)	166 (14.4)	139 (12.1)	31 (2.7)	8 (0.7)
Physical pain	Aching mouth	464 (40.3)	354 (30.7)	214 (18.6)	89 (7.7)	31 (2.7)
	Discomfort in eating food	439 (38.1)	291 (25.3)	291 (25.2)	100 (8.7)	31 (2.7)
Psychological discomfort	Being self-conscious	530 (46.0)	206 (17.9)	230 (20.0)	111 (9.6)	75 (6.5)
	Feeling nervous	600 (52.1)	206 (17.9)	213 (18.5)	82 (7.1)	51 (4.4)
Physical disability	Unsatisfactory diet	797 (69.2)	158 (13.7)	118 (10.2)	50 (4.3)	29 (2.5)
	Interrupting meals	651 (56.5)	268 (23.3)	160 (13.9)	44 (3.8)	29 (2.5)
Psychological disability	Embarrassed	609 (52.9)	275 (23.9)	181 (15.7)	63 (5.5)	24 (2.1)
	Difficulty relaxing	607 (52.7)	205 (17.8)	213 (18.5)	72 (6.3)	55 (4.8)
Social disability	Irritable with other people	645 (56.0)	202 (17.5)	195 (16.9)	69 (6.0)	41 (3.6)
	Difficulty doing usual jobs	773 (67.1)	201 (17.4)	118 (10.2)	45 (3.9)	15 (1.3)
Handicap	Life less satisfying	789 (68.5)	164 (14.2)	115 (10.0)	54 (4.7)	30 (2.6)
	Unable to function	790 (68.6)	181 (15.7)	119 (10.3)	38 (3.3)	24 (2.1)

**Table 3:** Association between combined OHIP-14 scores with self-rated oral health and pain or discomfort in the mouth^∗^.

Variable	*n* (%)	Mean (±SD)	*p* value
Self-rated oral health			
Poor	179 (15.5)	14.6 (3.7)	<0.001
Fair	108 (9.4)	13.3 (2.7)	
Good	396 (34.4)	13.9 (3.4)	
Very good	346 (30.0)	11.3 (2.7)	
Excellent	123 (10.7)	10.4 (3.2)	
Pain or discomfort in teeth or gum or mouth			
Never	303 (26.3)	11.1 (2.6)	0.012
Rarely	344 (29.9)	11.8 (3.1)	
Sometimes	372 (32.3)	12.8 (3.3)	
Often	133 (11.5)	13.4 (3.8)	

^∗^Kruskal-Wallis test.

**Table 4:** Relationship of the OHRQoL category with sociodemographic characters, lifestyle factors, and oral health behaviors.

Characteristics	Total sample (1152)	Poor OHRQoL		Binomial logistic regression	
		No	Yes	Adjusted OR (95% CI)	*p* value
Age (mean ± SD)	20.98 ± 1.9			0.958 (0.835–1.09)	0.533
Gender Female Male	558 594	470 (84.2) 485 (81.6)	88 (15.8) 109 (18.4)	Ref 1.89 (1.23–2.94)	0.004
College type Healthcare-related college Other colleges	490 662	396 (80.8) 559 (84.4)	94 (19.2) 103 (15.6)	Ref 1.35 (0.94–1.93)	0.108
Year of education First Second Third Fourth Fifth/intern	303 240 233 154 222	259 (85.5) 187 (77.9) 187 (80.3) 129 (83.8) 193 (86.9)	44 (14.5) 53 (22.1) 46 (19.7) 25 (12.7) 29 (13.1)	Ref 0.81 (0.65–1.35) 1.19 (0.67–2.11) 0.76 (0.58–1.36) 0.85 (0.53–1.41)	0.101 0.553 0.478 0.688
Smoking/shisha habits No Yes: daily Yes: rarely	928 156 68	785 (84.6) 116 (74.4) 54 (79.4)	143 (15.4) 40 (25.6) 14 (20.6)	Ref 3.47 (1.97–4.82) 2.25 (1.52–2.17)	<0.001 0.002
Carbonated drink consumption Never Seldom/rarely Once daily	152 599 401	125 (82.2) 488 (81.5) 342 (85.3)	27 (17.8) 111 (18.5) 59 (14.7)	Ref 0.621 (0.34–1.14) 0.971 (0.57–1.66)	0.128 0.916
Consumption of chocolates and candies Never Seldom/rarely Once daily More than once in a day	45 525 397 185	39 (86.7) 426 (81.1) 325 (81.9) 139 (75.1)	6 (13.3) 99 (18.9) 72 (18.1) 46 (23.4)	Ref 0.98 (0.75–1.74) 1.23 (0.84–1.98) 1.54 (1.10–2.19)	0.969 0.317 0.034
Brushing teeth per day Twice or more Once/never	468 684	403 (86.1) 552 (80.7)	65 (13.9) 132 (19.3)	Ref 0.78 (0.56–1.34)	0.601
Visit to the dental provider periodically (every 6 months) Yes No	460 690	408 (88.7) 545 (78.9)	52 (11.3) 145 (21.1)	Ref 2.23 (1.53-2.86)	0.002

Variable(s) entered on step 1: age in years, gender, college type, year of education, smoking status, carbonated drink intake, chocolates and candies consumption, brushing per day, and periodic dentist care.

## Data Availability

The data used to support the findings of this study will be provided from **the** corresponding author on request.
